# Microarray MAPH: accurate array-based detection of relative copy number in genomic DNA

**DOI:** 10.1186/1471-2164-7-163

**Published:** 2006-06-30

**Authors:** Brian Gibbons, Parikkhit Datta, Ying Wu, Alan Chan, John AL Armour

**Affiliations:** 1 Institute of Genetics, University of Nottingham, QMC, Nottingham NG7 2UH, UK; 2 PamGene International B.V., P.O. Box 1345, 5200 BJ 's-Hertogenbosch, The Netherlands

## Abstract

**Background:**

Current methods for measurement of copy number do not combine all the desirable qualities of convenience, throughput, economy, accuracy and resolution. In this study, to improve the throughput associated with Multiplex Amplifiable Probe Hybridisation (MAPH) we aimed to develop a modification based on the 3-Dimensional, Flow-Through Microarray Platform from PamGene International. In this new method, electrophoretic analysis of amplified products is replaced with photometric analysis of a probed oligonucleotide array. Copy number analysis of hybridised probes is based on a dual-label approach by comparing the intensity of Cy3-labelled MAPH probes amplified from test samples co-hybridised with similarly amplified Cy5-labelled reference MAPH probes. The key feature of using a hybridisation-based end point with MAPH is that discrimination of amplified probes is based on sequence and not fragment length.

**Results:**

In this study we showed that microarray MAPH measurement of *PMP22 *gene dosage correlates well with *PMP22 *gene dosage determined by capillary MAPH and that copy number was accurately reported in analyses of DNA from 38 individuals, 12 of which were known to have Charcot-Marie-Tooth disease type 1A (CMT1A).

**Conclusion:**

Measurement of microarray-based endpoints for MAPH appears to be of comparable accuracy to electrophoretic methods, and holds the prospect of fully exploiting the potential multiplicity of MAPH. The technology has the potential to simplify copy number assays for genes with a large number of exons, or of expanded sets of probes from dispersed genomic locations.

## Background

The role of submicroscopic DNA copy number variation in genetic pathologies has been established now for two decades [1]. Early investigations recognised the importance of deletions or duplications in specific genes as causative mutations in clinical conditions, such as *BRCA1 *in familial breast and ovarian cancer [2] and *DMD *in Duchenne/Becker muscular dystrophy [3]. Furthermore, subtelomeric chromosomal rearrangements leading to copy number changes have been associated with learning disability and other developmental abnormalities [4]. Recent advances in the diagnostic technologies applied to such conditions have also proven to be useful tools in elucidating the dynamic model of the human genome, with copy number variation recently recognised as an important component of human polymorphism [5-8].

As the significance of copy number variation in genetic analysis becomes more widely recognised so does the need to improve and extend the range of techniques available. In particular, for scanning multi-exon genes for deletions or duplications, MAPH and MLPA [9] have been used to assess the dosage of small (about 100 bp) regions to high accuracy (reliably discriminating 3 copies from 2), properties not easily implemented using, for example, current array-CGH methods. One feature shared by both Multiplex Amplifiable Probe Hybridization (MAPH) [10] and Multiplex Ligation-dependent Probe Amplification (MLPA) [11] is the limit to multiplicity set by the resolution of probes distinguished by their length, using capillary electrophoresis. In MAPH, the requirement for specific and stable hybridization sets a lower limit on probe size of around 100 bp, and the requirement for comparable co-amplification of different probes sets an upper limit of around 600 bp; thus the largest single probe set could contain 100 probes spaced 5 bp apart. While this does not pose a limitation on applications using relatively small probe sets, such as the 23 probes used in *BRCA1 *testing [12], where many exons are involved, such as *DMD *[13], or where screening is not targeted to single gene loci, probe sets of this magnitude would be inadequate.

The most obvious route to breaking these limits is to employ DNA microarray technology to replace capillary gel electrophoresis as the final analytical step in MAPH, as discussed in the original MAPH publication [10] and in a more recent review [14]. A microarray based on oligonucleotides complementary to individual MAPH probes would allow detection and quantification based on sequence rather than on size. Microarray detection and quantification should therefore allow the simultaneous measurement of copy number at a larger number of loci.

The initial stages of microarray MAPH, up to the recovery of specifically bound probes, remain the same as capillary MAPH. The subsequent amplification of these probes is modified to allow the incorporation of labels suitable for a standard two-channel photometric array analysis. Thus, Cy3-PZA primer is used for unknown samples and Cy5-PZA is used to generate a reference sample from a sub-population of normal males for comparison. The oligonucleotide spots of the array are co-hybridised with Cy3-probes from test samples and Cy5-probes from reference samples. Copy number can then be calculated by measuring the ratio of Cy3:Cy5 intensities, reflecting the amount of probe in the test (Cy3 labelled) samples relative to the reference (Cy5 labelled) samples. Thus, an elevated copy number would be revealed by a Cy3/Cy5 ratio greater than one, and less than one for a reduced copy number.

PamGene's flow-through microarrays use a novel format in which probe is actively pumped repeatedly across the plane of the array (hence "3-D"); diffusion is no longer the rate-limiting step, allowing faster hybridization kinetics than in diffusion-limited systems [15-17]. In this study we used the flow-through arrays in conjunction with the Olympus FD10 microarray instrument, which automates the required hybridization, pumping and image capture. Applications of the FD10 instrument in mutation detection and gene expression analyses have been published [18-21].

Using PamGene's 3-Dimensional, Flow-Through Microarray Platform we present a new format for MAPH: Microarray MAPH, using *PMP22 *gene dosage measurements in normal controls and CMT1A patients as an example. In this paper we demonstrate for first time successful combination of microarray technology with MAPH, and show that the accuracy of microarray-based MAPH is comparable to established electrophoretic methods.

## Results

Normalised data from microarray images were analysed in groups of normal and CMT1A samples for an initial global comparison of each endpoint of the mean returned copy number for each probe (Table 1). The CMT1A group has an elevated mean copy number for *PMP22 *probes, consistent with heterozygous duplication, in both microarray and standard capillary assays. The standard deviations for the majority of probes are similar for each format, suggesting that the microarray format reports the amounts of each probe as accurately as capillary electrophoresis. However, probes B1 and Da show unusually high standard deviations in the microarray MAPH, suggesting an additional source of variation in this format. High variation in reported dosage for probe B1 was traced to low spot intensity. Both the Cy3 and Cy5 intensities for each of the three B1 complementary oligonucleotides for all samples were similar to that of the non-human control probes (results not shown) and were thus no greater than background. This effect was independent of the oligonucleotide used, the position on the array and the sample analysed and therefore is probably due to an unusual property of this specific probe resulting in its poor capture on the array. We were, however, unable to identify obvious areas of self-complementarity in this sequence that might prevent capture by the array. In contrast, the high variation seen with probe Da is entirely due to two individual results that returned higher than expected intensities in the Cy3 images. These are most likely to be array image artefacts and are consistent with the tendency of Cy3 images to be more susceptible to substrate and dust particle fluorescence compared to Cy5 images, as can be seen to some extent in Figure 2.

The accuracy and precision of both formats, represented by the distribution of "MRD6" values (see "Methods") in the normal and CMT1A populations are very similar (Figure 3). This is particularly clear when directly comparing the mean MRD6 value and MRD6 standard deviation for each population shown in Table 2. The close agreement between the results of each format is confirmed by a correlation coefficient of 0.951 for MRD6 values for the full data set (Figure 4). We conclude that the PamChip microarray format for MAPH is able to determine copy number for *PMP22 *as accurately as the previous format using capillary electrophoresis.

## Discussion

In this study we have successfully demonstrated the combination of two powerful technologies, MAPH and the PamGene 3D Microarray. By taking the same source material through analyses with both capillary MAPH and array MAPH we have shown both endpoints to be in close agreement. This conclusion was based on three key observations. Firstly, each sample was correctly partitioned into one of two distinct populations on the basis of the MRD6 score: for normal samples the MRD6 ranged between 0.81 and 1.14; for duplicated (CMT1A) samples, MRD6 ranged between 1.20 and 1.71. Secondly, the average normalised copy number for each probe (with two artefactual exceptions) across the sample set was very similar. Thirdly, the MRD6 values for the two assays were closely correlated.

The potential for non-systematic noise in array MAPH was illustrated by the unusually high Cy3 intensity of two data points for spots complementary to the Da probe. These high intensity 'spikes' are caused by dust particles settling onto the array surface which tend to fluoresce more intensely in the Cy3 emission spectrum than in the Cy5. We did not take any particular precautions to ensure a dust-free environment for our experiments, and it is possible that these additional steps may have eliminated dust particles. Although it is doubtful whether dust contamination can be completely eliminated, especially when using the more open FD10, the more enclosed design of more recently released array instruments such as the PamStation 4 and PamStation 96 should also help reduce contamination. It is clear that applications where diagnosis may depend on the result of a single probe, such as the subtelomeric sets, it will be necessary to screen and control very carefully for spurious signals due to this kind of noise. For example, employing duplicate PCRs from samples, or duplicate spots on each array would distinguish false-positive signals from true copy-number change, and the data from this study show that signals from duplicate hybridizing spots have a correlation coefficient of 0.86, and a mean pairwise difference of about 11%. Thus gross anomalies (for example, due to dust particles) might be efficiently detected as poorly correlated signals, and removed from the data set. However, in order to present a clear picture of the potential of the system, the data presented in this report were analysed without any prior selection. As we have seen with probe B1 the potential for systematic noise also exists in array MAPH. As hybridisation is a key step of MAPH we can anticipate a high rate of success in using MAPH probe sets in microarray analysis without further modification, but it is unrealistic to expect that all probes will perform equally well. It is likely then that development of future applications of array MAPH may involve some minor modifications of existing probe sets.

In comparison with other established and commercially available technologies, there are some clear advantages and disadvantages to the technology we describe in this report. ROMA [7] uses competitive hybridization of test and reference "representations", made by amplification of restriction fragments, to arrays of oligonucleotides. Detection of putative copy number changes by allele-specific microarray methods [22-24], including recent work using Molecular Inversion Probes [25], depends on analysis of allele ratios from genome-wide SNP analyses.

Published work on copy number measurement using Molecular Inversion Probes [25] can involve not only information from allele ratios, but also from total signal ("copy sum"). The relative standard deviation from MIP "copy sum" analyses is of the order of 0.05–0.15 for most probes, but data analysis procedures including the removal of outliers, and "smoothing" procedures combining data from neighbouring loci, make it difficult to derive a fair comparison with the data presented here. "Smoothing" across neighbouring loci does improve the sensitivity, but at a cost of resolution. Furthermore, while in principle it is possible to use custom Molecular Inversion Probes based on invariant positions (and therefore using only "copy sum", not allele ratio measures), the published data makes use of established assays based on SNPs [25]. The resolution of MIP analyses is therefore limited by the spacing of the probes, but even if placed at very high density in custom sets, the requirement for data smoothing inevitably makes the effective resolution lower. Thus while MIP technology may have real advantages for truly genome-wide scans for copy number change, it is much less suitable for targeted analysis of (for example) exonic deletion and duplication in multi-exon genes, where the accuracy of single-probe measurement is critical.

MAPH has the advantage over MLPA [11], MIP and ROMA that MAPH results are not sensitive to unexpected substitutional variation in critical positions. In contrast to ROMA, MIP, and other allele-specific technologies which "smooth" data over neighbouring loci, the true effective resolution of MAPH is given by the size of the probe (100–500 bp). MAPH probes can be designed for nearly all single-copy sequences, so that a direct test can be made of very short sequences of interest (such as exons). By contrast, deduction of copy number changes from ROMA and allele-specific SNP arrays depends on indirect inference from the states of neighbouring markers, and the placement of these markers is dictated not by the biological question but by their inclusion in a subgenomic representation (in ROMA) or by the existence of common SNP variation (in allele-specific analyses). MAPH also analyses relatively small amounts (0.5–1 μg DNA) of genomic DNA without prior manipulation or amplification, both conserving genomic DNA resources and reducing the opportunity for distorting representation before analysis.

Nevertheless, for other well-established methods capable of full whole-genome copy number measurement, current commercial availability and the capacity to screen the whole genome in a single assay currently outweighs their limitations. Furthermore, in some analyses there may be a real advantage to allele-specificity, making methods like ROMA and MAPH less advantageous. For all these reasons, the particular advantages of microarray MAPH may make it most suitable for a targeted analysis of an intermediate number of loci at very high precision and resolution. Examples could include interrogating a set of 50–300 exons from selected "target" genes, or a set of 400–500 single-copy loci for analysis of karyotypic changes at relatively low resolution, but with high signal:noise ratios at each locus tested, and in a simple, fast and inexpensive assay.

MAPH is constrained by core elements of the assay, including probe sequence specificity, PCR efficiency and hybridisation kinetics. Probe design for capillary MAPH is further complicated by the electrophoretic end-point used to determine relative probe dosage. Removing these restrictions has implications for current and future applications of MAPH as an analytical tool. Probe sets can now be refined, if necessary, to allow simultaneous use of probes of similar lengths. One of the great strengths of MAPH, its multiplicity, has also been limited by the resolution of capillary electrophoresis. Probe sets that have been too large to be accommodated in a single gel run can now be combined to allow inexpensive and efficient analysis of larger numbers of loci simultaneously.

We chose to base array MAPH on PamGene's Flow-Through Microarray Platform. The superior hybridisation kinetics at the heart of the PamChip technology has shortened the hybridisation step from overnight to less than an hour. Even so, the implementation of the technology used here allows only four samples to be tested in the time it would take to resolve around thirty two samples on capillary analysis. Since this study was begun PamGene have developed the PamStation 96 which, as the name implies, increases the sample throughput of the technology via a 96-well format. In parallel with improvements in the PamChip technology we have also been successful in evolving the core MAPH protocol into a 96-well format. Together, these advances have the potential to unlock the true power of MAPH in combination with PamGene's microarray systems.

## Conclusion

We have been able to use oligonucleotide microarrays to display the products from MAPH for the analysis of DNA copy number. The accuracy of the copy number determinations compares favourably with results obtained with standard electrophoretic separation of fluorescently-labelled fragments. Microarray readout removes the upper limit imposed by electrophoretic mobility on the multiplicity, and in principle will allow the extension of MAPH as a highly accurate, high-resolution method for simultaneous copy number determination at larger numbers of loci.

## Methods

### Sample material

As microarray MAPH differs from capillary MAPH only in its final stages we were able to re-examine primary MAPH products produced in a previous study of the application of MAPH to *PMP22 *gene dosage measurements [26]. This material has already been subject to capillary electrophoretic MAPH analysis confirmed by clinical diagnosis and so provided an ideal basis for the direct comparison of the two techniques. For this study we selected a total of 49 primary MAPH samples representing 38 individuals, of which 28 samples (26 patients) were confirmed normal and 21 samples (12 patients) were confirmed CMT1A, with a heterozygous duplication of *PMP22*. To ensure DNA integrity, these preparations were freshly amplified and analysed following the established capillary MAPH protocol.

### PMP22 probe set

DNA sequences for the *PMP22 *probe set and for other MAPH probes along with protocols for probe set and probe mix preparation can be found at [27]. The set consisted of six probes from the target *PMP22 *gene and nine reference probes from unlinked autosomal loci, together with two sex linked probes and one non-human probe that together acted as controls for specificity of washing and hybridisation.

### PamChip microarray design

Complementary 60 mer oligonucleotides for each probe were initially designed as close as possible to a nucleotide composition of 50% G/C (oligonucleotides "A"). To help with the development process of the new format, two additional complementary oligonucleotides for each probe were designed allowing more variation in the GC content. Thus, each probe had three complementary oligonucleotides designated "A", "B" and "C", with "A" designed to be as close as possible to 50% G/C. Analysis of the data from individual array spots (data not shown) revealed a lower GC limit of 40% below which probes either completely failed to be captured or showed evidence of hybrid instability via highly variable spot intensities. Nevertheless, initial analysis suggested that most consistent results were obtained by using all the complementary spots for each probe ("A", "B" and "C") to generate an average spot intensity. In addition to testing three oligonucleotides per probe, the entire array was spotted in duplicate giving a total of six array spots for each probe.

### Cy5 reference

To produce the Cy5-labelled reference sample a number of MAPH primary products from five normal male samples were individually amplified by PCR using Cy5-PZA. These were purified and pooled to give a single homogenous reference DNA source.

### Capillary MAPH

Full experimental details of MAPH have been published previously [10,26,28] and updates to the protocol are available at [29]. In this case each MAPH hybridisation-selected primary product was amplified by PCR with Cy3-PZA so that the same labelled probes could be analysed by capillary and microarray MAPH. Amplified products were purified and then resolved electrophoretically using the ABI Prism 3100 Genetic Analyser with a 36 cm capillary array loaded with POP-4™ polymer. An injection time of 40s produced peak areas for all probes that were within the sensitive range of the instrument. Electrophoretograms were integrated and peaks sized using a combination of ABI Prism's Genescan and Genotyper software. Peak areas were used to calculate relative probe dosage as previously described [28].

### Microarray MAPH

**Figure 1:**
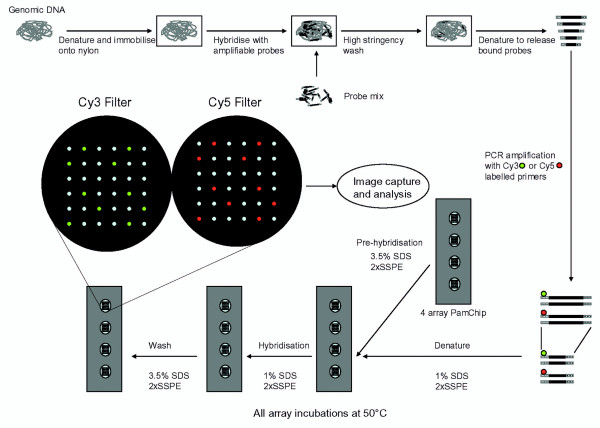
Overview of microarray MAPH. Amplifiable probes are hybridised with immobilised and denatured genomic DNA, and after stringent washing, specifically-bound probes are recovered. After PCR amplification, recovered probes are specifically detected and quantified by hybridization to oligonucleotides on the microarray.

An overview of the array MAPH protocol can be seen in Figure 1. Co-hybridisation mixtures were composed of 8 μl of Cy3 labelled probe solution, 8 μl of Cy5 labelled reference probe solution, 2 μl of 10% SDS and 2 μl of ×20 SSPE and were held on ice until needed. Before being applied to the array, this mixture was denatured (>95°C, 5 minutes). PamChips were loaded into the Olympus FD10 microarray instrument according to the manufacturer's instructions. A custom protocol was designed around a 50 μl displacement volume, 5 μl/s flow rate, and 5s hold after each half cycle of the pump. Each incubation involved a pre-hybridisation stage of 2 repeats of 15 cycles, a single stage of 60 cycles for the hybridisation, and 2 repeats of 5 cycles for post-hybridisation.

### Copy number determination

**Figure 2:**
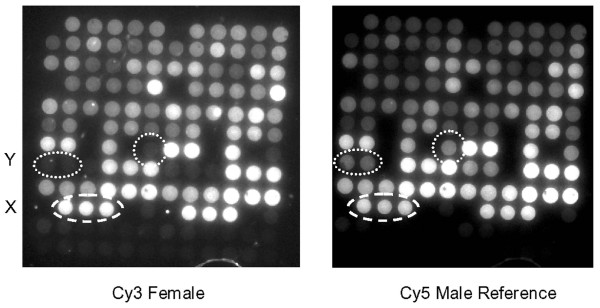
Array MAPH images. Typical image-captures of a single array from a *PMP22 *PamChip after co-hybridization with a Cy3 labelled female patient sample (left) and the Cy5 labelled male reference sample (right). Spots containing oligonucleotides complementary to the Y and X chromosomes are indicated. The 12 × 12 arrays shown are composed of duplicate spots of 72 oligonucleotides (in 6 columns and 12 rows), such that the left and right halves of the array are identical. The oligonucleotides occupying the bottom two rows are control sequences not complementary to the probes applied.

**Figure 3:**
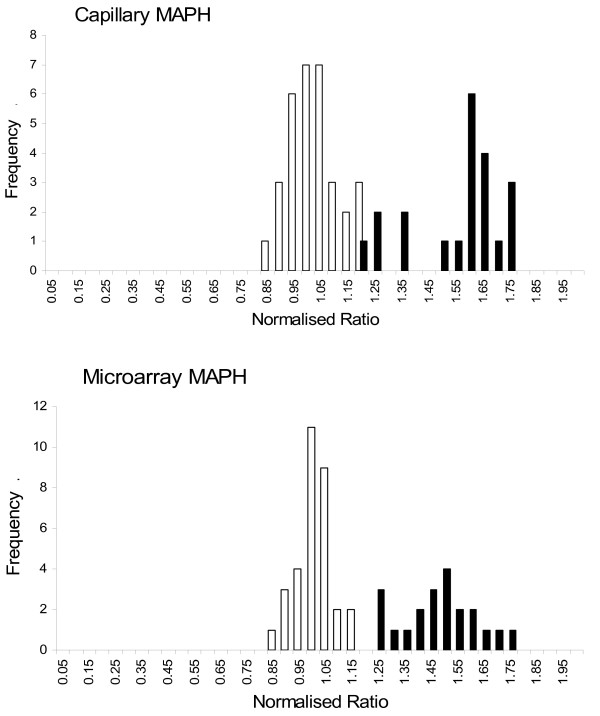
Distributions of copy number scores, comparing capillary and microarray MAPH. Histograms of values for normalised copy number, using the "MRD6" values described, for measurements using capillary MAPH (upper) or microarray MAPH (lower). White bars show the normal group and black bars the duplicated (CMT1A) group.

**Figure 4:**
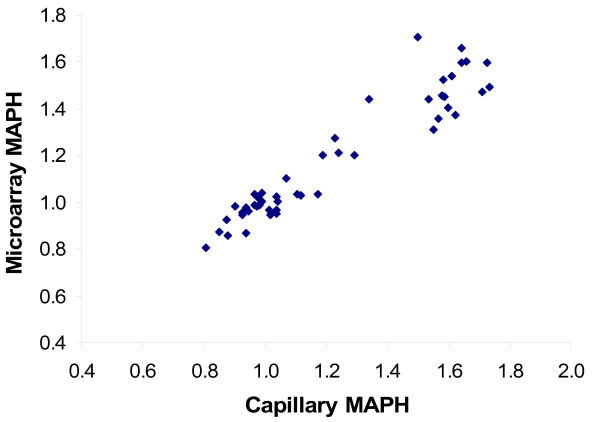
Correlation of MRD6 copy number measures on the same samples between capillary MAPH and array MAPH. The value of the correlation coefficient is 0.95.

Images of Cy3 and Cy5 intensity were captured in 12 bit greyscale TIFF format using manual control of the proprietary software pre-installed on the FD10. Example image captures from a female sample co-hybridised with the male reference are shown in Figure 2. Captured images were then loaded into PamChip Analyser (V.4) which was set to record values for median spot intensity minus a background value for each spot using the 'local corners' method. The tabulated spot intensity data was exported directly into Microsoft Excel 10 where normalised ratios of Cy3/Cy5 were calculated. Initial Cy3/Cy5 ratios were calculated using the average intensity from all six spots for that probe and then processed through two levels of normalisation. The first level of normalisation addressed inter-array variation in overall image intensity principally brought about by differences in sample DNA concentration. By assuming an expected ratio of 1/1 for autosomal control probes in each sample all ratios returned for that sample can be adjusted to the mean of these probes.

A second level of normalisation was used to address differential PCR amplification efficiency of individual probes between sample and reference amplifications, and adjusted the ratio for individual probes to the mean ratio among unaffected controls. Copy number for each probe was then expressed as this final normalised ratio. The definitive *PMP22 *dosage was determined as the mean relative dosage of the six *PMP22 *probes, the so-called 'MRD6' value [26].

Ratios for the Y-linked probe were normalised to the mean ratio of male samples only. Ratios for the X-linked probe were normalised to the sum of the X-linked probe ratios divided by half the total number of X chromosomes present in the test population, i.e. by normalising to female genome equivalents.

## Authors' contributions

The study was generally planned and organised by AC and JA. The experimental work was performed by BG and PD in Nottingham, with planning, supervision and coordination by JA, and by PD at PamGene, with planning, supervision and coordination by YW and AC. Materials, equipment and expertise from PamGene were coordinated by AC and YW. Experimental data were analysed and interpreted by BG, PD and JA. The manuscript was initially drafted by BG and JA; all authors read the manuscript and made comments on it; and JA organised the final versions of the manuscript, tables and figures and arranged submission.

## Figures and Tables

**Table 1:** Comparison of capillary MAPH and microarray MAPH of 28 normal samples and 21 CMT1A samples. The upper group of results show the mean and standard deviations for normalised data separated according to probe category and known pathology. * Medians used rather than means to reduce distortion by extreme outliers; see "Results" for further details.

	Capillary MAPH				Microarray MAPH			
PMP22 Probes	Normal (n = 28)		CMT1A (n = 21)		Normal (n = 28)		CMT1A (n = 21)	
	
	Mean	SD	Mean	SD	Mean	SD	Mean	SD

PMP1A	0.986	0.081	1.490	0.183	1.000	0.082	1.411	0.195
PMP4	0.986	0.089	1.567	0.237	0.982	0.082	1.490	0.216
CMT5	0.962	0.127	1.607	0.290	0.981	0.161	1.428	0.210
CMT10	0.980	0.120	1.597	0.241	0.980	0.108	1.466	0.247
PMP2	0.994	0.075	1.449	0.131	0.991	0.073	1.450	0.117
PMP3	0.972	0.098	1.471	0.101	0.973	0.132	1.420	0.149

PMP22 Mean	**0.980**	0.098	**1.530**	0.197	**0.985**	0.106	**1.444**	0.189
	
	Capillary MAPH				Microarray MAPH			

Autosomal Probes	Normal (n = 28)		CMT1A (n = 21)		Normal (n = 28)		CMT1A (n = 21)	
	
	Mean	SD	Mean	SD	Mean	SD	Mean	SD

ST8D2 (19p	0.966	0.087	1.002	0.093	0.991	0.106	0.936	0.109
ST8B4 (18q)	0.960	0.117	1.058	0.161	0.991	0.074	1.014	0.094
B1	1.025	0.070	0.979	0.076	1.260*	0.811	0.970	0.169
ST10b4 (7p)	0.994	0.082	0.957	0.064	1.004	0.108	0.963	0.064
Da	1.012	0.113	0.974	0.091	1.138*	0.438	1.018	0.181
ST5C3 (8p)	0.997	0.118	1.112	0.172	1.031	0.088	1.070	0.120
ST4a4 (2q)	1.000	0.057	0.975	0.078	0.992	0.060	0.973	0.089
ST11G9 (19q)	0.989	0.160	0.968	0.099	0.952	0.123	0.957	0.153
A2	0.960	0.135	1.071	0.164	1.000	0.285	1.050	0.175

Autosomal Mean	**0.989**	0.104	**1.011**	0.111	**1.040**	0.233	**0.994**	0.128
	Male		Female		Male		Female	
	Mean	SD	Mean	SD	Mean	SD	Mean	SD
Y	0.522	0.061	0.021	0.014	0.530	0.070	0.083	0.055
X	0.482	0.077	0.952	0.424	0.555	0.107	0.930	0.312

**Table 2:** "MRD6" copy number score variation for capillary MAPH and microarray MAPH.

	**Normal**			**CMT1A**		
	Mean	SD	%	Mean	SD	%

Capillary MAPH	0.980	0.092	9.377	1.530	0.168	10.995
Microarray MAPH	0.985	0.072	7.318	1.444	0.147	10.179
